# Irrigation Solutions in Total Joint Arthroplasty

**DOI:** 10.51894/001c.37502

**Published:** 2022-09-06

**Authors:** Matthew Caid, Josiah Valk, Jonathan Danoff

**Affiliations:** 1 Orthopedic Surgery Beaumont Health; 2 Orthopedic Surgery Northwell Health

**Keywords:** prosthetic joint infection, taurolidine, hydrogen peroxide, acetic acid, chlorhexidine, povidone-iodine, total hip arthroplasty, total knee arthroplasty, irrigation solution

## Abstract

**INTRODUCTION:**

Despite advancements in the field of adult reconstruction, prosthetic joint infection (PJI) remains a common and devastating complication of total joint arthroplasty. Eradication of these infections can often prove difficult, and they remain a source of considerable morbidity and mortality. This clinical review paper will focus on some of the more commonly used irrigation solutions; povidone-iodine (PI), chlorhexidine (CHG), acetic acid (AA), hydrogen peroxide (HP), antibiotic irrigations, taurolidine, and polyhexanide-betaine (PB)

**SUMMARY OF THE EVIDENCE:**

Significant research has been performed on the prevention of PJI, including use of intraoperative joint irrigation solutions. Several solutions have been theorized to aid in infection prevention, but no evidence-based practice guidelines in this area of orthopaedics have been established. There is a paucity of prospective randomized control trials to compare the efficacy of these joint irrigation solutions.

**CONCLUSIONS:**

The authors present a review regarding seven major categories of commonly used intraoperative joint irrigation solutions. The current literature fails to demonstrate a clear consensus for a preferred solution and concentration for povidone-iodine, chlorhexidine, hydrogen peroxide, acetic acid, antibiotic irrigations, taurolidine, and polyhexanide-betaine. Prospective, randomized control trials directly comparing these different irrigation solutions are needed.

## INTRODUCTION

Infections after total hip and total knee arthroplasty can be extremely costly to the healthcare system in addition to carrying significant patient morbidity and mortality.^1^ The current incidence of prosthetic joint infection (PJI) has been reported to be approximately 1-3%.^1,2^ Although this is an overall infrequent complication, the total number of future infections are projected to grow due to the exponential increase in total joint replacement that is expected over the next decade.^3^ While many complications in total knee arthroplasty (TKA) are decreasing, comparatively, the incidence of PJI remains unchanged.^4^ The prevention and treatment of PJI is a pressing priority in the field of arthroplasty. Treatment for PJI can be quite morbid- requiring multiple surgeries and time off work. Joint infections also decrease patients’ quality of life and can lead to mortality.^5^

Subacute and chronic PJI are particularly difficult to eradicate due to development of biofilm. Biofilms are protective, microscopic, three-dimensional environments for bacteria that are encased by an outer exopolysaccharide layer.^1^ Mature biofilm is present 3-6 weeks after inoculation and forms on native tissue, metal components, cement, and bone.^1^ There are four stages of biofilm formation; attachment, cell aggregation, biofilm maturation, and detachment, where bacterial cells are shed and act as a nidus for further infection and biofilm propagation.^6^ Biofilm protects the bacteria from antibiotics, antiseptics, and the host immune response.^7^

No current technique has been proven to remove biofilm from arthroplasty components. Pulse lavage with normal saline does not eradicate biofilm from TKA components; this was confirmed by using bioluminescent imaging in-vitro.^8^ Explantation of the arthroplasty components and a thorough debridement with synovectomy is the only known method to definitively clear the infection once a biofilm has formed.^1^ Single-stage and two-stage revision arthroplasty are both viable treatment options.^9^ Currently, in North America, a two-stage revision is the more commonly utilized technique.^9^

The prevention of PJI has been well-studied, including publications on decreasing operating room traffic, perioperative antibiotic administration, effect of laminar flow operating rooms, and patient optimization. Intraoperative irrigation during total joint arthroplasty remains an intriguing target. To date, several different irrigation solutions have been studied. This paper will focus on some of the more commonly used solutions; povidone-iodine (PI), chlorhexidine (CHG), acetic acid (AA), hydrogen peroxide (HP), antibiotic irrigations, taurolidine, and polyhexanide-betaine (PB).

These solutions are used intraoperatively to theoretically decrease the bacterial burden in the surgical wound prior to closure. The primary goal is to decrease the number of postoperative infections. Solution efficacy must be weighed against safety as these solutions have varying cytotoxic effects on native human tissue.^10–17^

Postoperative infections are not a complication isolated to joint arthroplasty and research into antiseptic irrigation solutions is on-going in many different fields.^18^ Without validated, head-to-head, randomized controlled trials- it is difficult to prove superiority of any given irrigation solution. The purpose of this paper is to review, condense, and consolidate the available research on various total joint arthroplasty irrigation solutions.

## SUMMARY OF THE EVIDENCE

### Povidone-Iodine

Povidone-iodine is a chemical complex of povidone and triiodide. It is an effective antiseptic with research in multiple surgical subspecialties including urology, plastic surgery, and general surgery.^18^ Aqueous povidone-iodine is bactericidal. Free iodine radicals released from povidone polymer complexes form an iodine solution that interferes with critical bacterial structures, including cell membranes and cytosolic enzymes. It also oxidizes nucleotides and amino acids.^19^ In contrast to various antibiotic and other antiseptic irrigations, PI not only destroys bacteria, but also inhibits the release of cell toxins and tissue-destroying enzymes. Resistance to PI has not yet been reported.^20,21^

The concentration and duration of exposure to PI must be taken into consideration. There is a hypothetical risk of iodine absorption into the bloodstream causing systemic illness. A systematic review of level I and level II studies concluded that PI was safe and effective as an intraoperative irrigation to prevent surgical site infections.^22^ It failed to demonstrate an increase in postoperative serum iodine. There were no observed patient complications. Notably, 10 of the 15 studies demonstrated that PI was significantly more effective at preventing surgical site infections (SSI) than saline, water, or no irrigation.^22^

The chondrotoxic effects of PI are pertinent in the setting of partial knee replacements and unresurfaced patellae. Povidone-iodine is recommended to be diluted with saline to a concentration of 0.35% and used as a soak or lavage in the wound for three minutes.^10–12^

There is some evidence to suggest povidone-iodine efficacy in animal and in-vitro studies. In one study, rabbit knees were implanted with stainless steel and polyethylene components, and an acute *Staphylococcus aureus* infection was induced.^23^ The knees were then irrigated with either PI or normal saline. The study reported a significant decrease in the bacterial load in those knees irrigated with PI when comparing the PI and saline cohorts. Cichos et al. directly compared the antiseptic activity of povidone-iodine, Chlorhexidine, and vancomycin powder against seven different bacteria from titanium discs.^24^ The following antiseptics were used; 1% PI, 0.05% CHG, and 5 ug/mL Vancomycin.

These different antiseptics were exposed to the bacteria coated titanium discs for zero-, three-, 30-, and 60-minute intervals. Povidone-iodine was the only agent able to kill all seven bacteria at all time intervals including zero minutes. CHG was unable to kill methicillin-resistant *Staphylococcus aureus* at zero and three minutes. This study concluded that PI was superior to CHG and vancomycin in this setting.^24^

Povidone-iodine is also efficacious in eradicating methicillin-resistant *Staphylococcus aureus* (MRSA). This virulent strain remains one of the most difficult infections and biofilms to eradicate. In-vitro studies show that PI killed all isolates of MRSA within 30 seconds.^25^

Of all the irrigation solutions, PI has the highest volume of published data in the field of arthroplasty. Following dilute 0.35% PI lavage before wound closure for primary TKA and total hip arthroplasty (THA), postoperative prosthetic infections in the first 90 days dropped significantly from 0.97% to 0.15%, p = 0.04.^12^ A randomized control trial of 457 patients undergoing primary TKA and THA compared the development of PJI in PI irrigation versus saline control. The study utilized dilute PI lavage soak in addition to painting the skin edges at closure with 10% povidone-iodine. The control group used 1L of normal saline irrigation. There were eight infections (3.4%) in the saline group and only one infection (0.4%) in the povidone-iodine group, p = 0.038.^10^

Not all publications support the use of PI. Large cohort retrospective analysis of joint registry data of 11,738 primary, and 2,884 revision TKA and THA failed to show a statistically significant benefit in using PI to prevent infection.^26,27^ The primary outcome was rates of reoperation due to infection at three- and 12-months status-post arthroplasty.

Overall, although reports on efficacy remain mixed, dilute PI lavage is relatively inexpensive with minimal risk of adverse effects.^10–12^ This has led to its recommendation from several organizations to prevent infection. The International Consensus Meeting Clinical Practice Guidelines in 2017 endorsed PI lavage. Other organizations such as the Center for Disease Control and the World Health Organization have given similar recommendations.^28–30^

### Chlorhexidine

Chlorhexidine (CHG) is a biguanide (i.e., a Nitrogen and Hydrogen-based organic) compound that exerts its antimicrobial effects by disrupting cell membranes and their contents.^31,32^ CHG is known for having broad-spectrum antibacterial coverage, persistent efficacy, and residual activity due to its high affinity for binding to skin and mucous membranes.^31,32^ For these reasons, it is commonly used for surgical site prepping, hand washing, dental procedures, and a wide range of disinfectant purposes. The Food and Drug Administration (FDA) approved a surgical irrigation composed of CHG and sterile water (Irricept, Irrimax).

Animal studies in cows and rats have not shown any wound healing compromise, loss of soft tissue strength, or decreased ability to produce healthy collagen.^33,34^ However, prolonged exposure to CHG at high concentrations can have deleterious effects.^13,14^ Case reports of six native knees that mistakenly had arthroscopies performed using 1% CHG instead of normal saline have been reported.^13^ The resultant outcomes were persistent pain, decreased range of movement, and loss of joint space in all three compartments.^13^

Additionally, an in-vitro study reported that compared to eight other irrigations, CHG was found to be the most cytotoxic irrigation for fibroblasts (i.e., connective tissue formation), chondrocytes (i.e., cells responsible for the formation of cartilage), and osteoblasts (i.e., bone development).^14^ No known studies have proven CHG to be harmful to humans at concentrations of 0.05%. However, until further safety studies have been performed, it should continue to be used with caution.

Chlorhexidine is also efficacious in the treatment of MRSA. Irrigation solutions were compared in their ability to decrease in colony forming units (CFU) of MRSA. Titanium discs were coated in a MRSA biofilm, and then scrubbed with saline, bacitracin and saline, castile soap, povidone- iodine, and chlorhexidine. They concluded that CHG was superior at decreasing CFU.^35^ The same model was then used to examine which concentration of CHG would be most effective at decreasing CFUs. They found that 2% CHG was the superior concentration.^36^ However, during this study the biofilm was not completely eradicated. This would limit its utility in treatment of PJI as arthroplasty components with a persistent bacterial biofilm will likely see the infection recur at some point.^37^

Potential synergistic effects of CHG and hydrogen peroxide have been investigated with *Staphylococcus aureus* and multiple *Streptococcus* species. Steinberg et al. exposed bacteria to CHG, Hydrogen Peroxide, and CHG plus HP, then mean inhibitory concentrations were measured. The authors found the combination of CHG and HP killed bacteria at a lower concentrations than either solution alone.^38^

Chlorhexidine gluconate has some evidence suggesting efficacy against biofilm production. One study investigated the ability of commonly used antibacterial and antiseptic solutions to disrupt biofilm.^15^ They exposed the bacteria to multiple concentrations of CHG (0.025%, 0.05%, 0.1%), PI (0.35%, 1.0%, 3.5%, 10%), sodium hypochlorite (0.125%, 0.25%, 0.5%), and triple antibiotic solution (bacitracin 50,000U/L, gentamicin 80 mg/L, polymyxin 500,000 U/L) all for one, five, 10 minutes, in triplicate. Failure to eradicate all bacteria was defined as growth in any of the three replicates at 21-day culture. Results demonstrated 0.05% and 0.1% CHG were the most effective at all exposure times. Only 10% povidone-iodine was effective at all time intervals. All other solutions failed at all concentrations and exposures.^15^

A retrospective review of a single surgeon’s protocol with CHG (0.05%) lavage vs PI (2%) was unable to identify a significant difference in infection rate.^39^ There were no significant differences regarding wound healing, superficial surgical site infections, and PJI. Further studies are warranted to help identify a superior solution, concentration, and duration of irrigation.

### Acetic Acid

Acetic acid is a colorless, organic liquid. It is one of the main components of vinegar. It has many uses including as a food preservative and as an antiseptic. Knowledge of its antiseptic properties’ dates back thousands of years. Hippocrates, the “Father of Medicine” (c. 420 BC), described using AA for wound healing and to treat infections.^40^ Acetic acid is a weak organic acid that can be effective against gram-negative and gram-positive bacteria.^41^ It is commonly used to treat burn victims by applying it topically or with acetic acid-soaked gauze at concentrations of 1-5%. *Pseudomonas aeruginosa* is a common biofilm in burn wounds that is particularly susceptible to acetic acid.^37^ Acetic acid has also been used to treat otitis media (i.e., middle ear infection), urinary tract infections, soft tissue ulcerations and wounds.^40^

Studies suggest the potential of AA to eradicate biofilms. One study investigated the efficacy of AA concentrations, ranging from 0.16-0.31%, in their ability to eradicate common bacterial biofilms that present in patients with burn wounds. All 29 biofilms which included gram negative and gram-positive bacteria were eradicated within 3 hours.^41^ Tsang et al. found quicker biofilm eradication at increasing AA concentrations with minimum biofilm eradication concentration being 15%, 11%, 3.2%, and 0.8% following 10-minute, 20-minute, 180-minute, and 24-hour treatments.^16^ There is concern for corrosive effects on biological tissues at AA concentrations exceeding 5%. However, at 3% and 5% concentrations they reported a biofilm eradication of 85.9% and 96.1% respectively. Due to these findings, the authors hypothesized a benefit to using AA in the revision arthroplasty setting.^16^

There is a paucity of literature on AA as an irrigation solution. One safety study examined 23 patients undergoing revision TKA who were treated with a 20-minute acetic acid (3%) bath. The revision procedures ranged from single stage, two stage, debridement antibiotics and implant reimplantation, and arthrodesis (i.e., fusion of adjacent bones). Three of the 23 patients (13%) were re-infected at 18 month follow up. They did not comment on re-infection data due to lack of standardization of procedure and the high reinfection rate. During the 20-minute AA bath, the patients remained hemodynamically stable.^42^ The high reinfection rate of 13% does not indicate a clear benefit of using AA. Additionally, a 20-minute irrigation bath is not practical intraoperatively, as other irrigation solution recommendations range from one to three minutes.

### Hydrogen Peroxide

Hydrogen peroxide is a pale blue liquid compound that acts on bacteria though disruption of deoxyribonucleic acid (DNA) synthesis through oxidation of proteins and membrane lipids.^32,43^ There are many theoretical benefits of its use as an antiseptic. Additionally, HP can help decrease bacterial load when used synergistically with other antiseptic agents.

A combination HP and PI irrigation protocol during single stage revision arthroplasty has been reported.^44^ Their protocol consisted of 12L of saline irrigation followed by irrigation with 200mL of 3% hydrogen peroxide followed by 200mL of 1% povidone-iodine, in addition to antibiotic cement and antibiotic pellets. They reported zero recurrence of infection in their 11 hips and 28 knees at five and six-year follow-up. These results support the synergistic effect of povidone-iodine and hydrogen peroxide. The increased efficacy is attributed to the effervescence (i.e., formation of “bubbles”) of HP which is able to mechanically disturb the biofilm, allowing for the PI to work more efficiently.

Additional in-vitro studies have suggested potential synergy with iodine and hydrogen peroxide. Three solutions, HP alone, PI alone, and HP in conjunction with PI, were applied to cultivated bacteria and yeast. When given separately, the solutions were bacteriostatic in three bacterial and 16 yeast species. However, when given in combination, the solutions were bactericidal.^45^

The dental literature boasts similar synergy with HP and CHG. When used in combination, these two antiseptics were more effective in killing *Staphylococcus faecalis* and *Staphylococcus sobrinus* in a test tube environment.^38^ Chlorhexidine gluconate is thought to damage the cell wall of bacteria allowing HP easier access and enhancing its ability to disrupt DNA synthesis.^32^

When examining the literature, it is common to see HP in combination with other antiseptics, but isolated use in clinical practice is scarce. Spine literature supports the use of combination HP irrigation. In one study of approximately 1,000 surgical cases, SSI rates were compared between patients treated with HP/PI combined irrigation versus a no irrigation control. The irrigation protocol consisted of 10mL of 10% PI, 1mL of 3% HP, and 5mL of sterile water. The surgical site was flooded with solution, and after one minute the solution was washed out by copious normal saline to minimize the risk of toxicity. Zero infections out of 490 cases were reported in the HP/PI group, compared to seven deep infections (1.5%) reported out of the 460 cases in the control group.^17^

Concerns regarding possible side effects of hydrogen peroxide include wound healing complications, toxicity to tissues, and embolic (i.e., blocking of an artery) events. Hydrogen peroxide is contraindicated in spinal surgery in the setting of dura (i.e., layer of connective tissue of the meninges of the brain) tears.^17^ One mL of hydrogen peroxide can generate 10mL of oxygen, creating a space occupying hazard, and posing a risk for air embolism.^46^ This raises safety concerns during the cement pressurization of a femur during total hip arthroplasty. There are several case reports in the THA literature that describe HP irrigation of femoral canals before cement pressurization followed by immediate cardiac arrest, attributed to the development of an air embolism.^47^ The use of hydrogen peroxide as a standalone orthopaedic irrigation is not supported in the literature; however, HP in combination with PI or CHG is promising and deserves further study.

### Antibiotic solutions

Perioperative intravenous antibiotics are paramount in the prevention of infection in arthroplasty. Cephalosporins are the “gold standard” recommended agent by the American Academy of Orthopaedic Surgeons.^48^ Vancomycin also has its role in the setting of penicillin anaphylaxis or a history of multidrug resistant infections.^49^ Antibiotic solutions for irrigation have been analyzed across many different specialties. Despite many authors’ interests, no study has successfully proven the ability of antibiotics solutions to prevent PJI. Ideally, surgeons could select antibiotic solutions based on known or suspected pathogens.

Goswami et al. compared the efficacy of eight different irrigation solutions including: povidone-iodine 0.3%, CHG 0.05%, saline 0.9%, vancomycin 1g/L, gentamicin 80 mg/L, castile soap 0.45%, polymyxin 500,000 U/L/bacitracin 50,000 U/L. *Staphylococcus aureus* and *Escherichia coli* broths were primed on 96-well plates and exposed to each irrigation for time intervals of one and three minutes. Bactericidal activity was measured alongside cytotoxicity to human fibroblast, osteoblast, and chondrocyte cells.

The study reported bactericidal inferiority of polymyxin/bacitracin to povidone-iodine and CHG.^14^ The polymyxin-bacitracin solution was ineffective at eradicating *Staphylococcus aureus* and *Escherichia coli.* Conversely, povidone-iodine and chlorhexidine proved to be effective against both pathogens. However, chlorhexidine demonstrated statistically significant cytotoxicity, 49.38 +/- 0.80%, p < 0.0001, to all three human cells tested when compared to the other solutions. Their triple antibiotic solution of bacitracin 50,000U/L, gentamicin 80 mg/L, polymyxin 500,000 U/L failed to demonstrate efficacy against biofilms at all concentrations and exposures. Exposures were for one, five, and 10 minutes. Failure to eradicate all bacteria was defined as growth in any of the three replicates at 21-day culture.^15^

Anglen et al. investigated the efficacy of various antibiotic irrigations at decreasing the number of bacteria left on orthopaedic screws. A high-powered spray consisting of bacitracin, neomycin, saline, and soap were blasted on 3.5mm orthopaedic screws coated in different bacterial species. The screws were then sent for sonication to evaluate how much bacteria were remaining. Bacitracin and neomycin proved no better than saline. Saline has been proven clinically ineffective at removing bacterial biofilm.^50^ Soap was superior to all solutions.^50^ However, all solutions were an improvement compared to the control group which consisted of bacteria-coated screws with no intervention.

Orthopaedic trauma literature also demonstrates no benefit of antibiotic irrigation. Anglen published a prospective randomized study comparing the use of bacitracin solution versus a non-sterile castile soap solution in patients with lower-limb open fractures. No statistically significant difference was demonstrated between groups when comparing infection rates, p = 0.2. However, the author did report a possible risk of wound healing complications in the bacitracin group.^51^

A meta-analysis of wound irrigation solutions utilized in general surgery analyzed 21 randomized controlled trials, consisting of intraoperative intraperitoneal, mediastinal, and incisional wound irrigation. The primary outcome was surgical site infections. The different antibiotics consisted of cefamandole nafate, tetracycline, gentamicin with clindamycin, ampicillin, cefotaxime, and cephradine. The non-antibiotic irrigations included povidone-iodine, saline and taurolidine. The authors concluded that there was no significant decrease in infection rate when using any of the antibiotic solutions in the abdomen, mediastinum, or incisional wounds. They did report that povidone-iodine showed significant benefit in reducing SSI.^52^

### Taurolidine

Taurolidine is a synthetic product derived from the aminosulfonic acid, taurine. The active metabolites of taurolidine bind to the bacterial cell wall. Chemically reactive hydroxymethyl groups occupy binding sites to induce damage to the cell wall of the bacterial surface in gram positive and gram-negative bacteria such as *Escherichia coli* and *Streptococcus pyogenes*.^53^ Taurolidine has antimicrobial and antifungal properties and has been widely used for sealing central line catheters and hemodialysis catheters to prevent infection and bacteremia. It has also been used clinically to treat peritonitis. Further studies are also examining its anti-neoplastic (i.e., ability to prevent tumor growth) properties.

Taurolidine was studied in a retrospective review of 300 patients irrigated with 2% taurolidine during a TKA versus a control group of 300 TKA patients who did not receive irrigation. The authors compared postoperative C-reactive protein (CRP) and erythrocyte sedimentation rates (ESR) as well as infection rate in both groups. The taurolidine group did not show any significant difference in CRP or ESR. The single TKA infection seen in the 600 patients occurred in the taurolidine group.^54^ The results of this study do not support the use of taurolidine to prevent infection in hip and knee arthroplasty.

### Polyhexanide-Betaine

Polyhexanide-betaine is a combination product used as an irrigation solution and wound care solution. It is available in a 0.1%/0.1% combination product (Prontosan, Braun). Polyhexanide is a broad-spectrum antiseptic and preservative that interferes with the bacterial cell membrane and leads to disruption via increased permeability.^55^ Betaine is a surfactant, which reduces surface tension and aids in debridement.^55^

While PB has shown efficacy for biofilm eradication in wound care, there remains a paucity of literature on in-vivo use for total joint arthroplasty. Davis et al. demonstrated MRSA biofilm eradication in porcine wounds with 97.85% and 99.64% bacterial load reduction at 3- and 6- day intervals.^56^ An in-vitro study of kill time for different planktonic bacterial cells revealed PB time to eradication of 90 seconds for *Staphylococcus aureus* and *Staphylococcus epidermidis* and 120 seconds for *Cutibacterium acnes*.^57^ Polyhexanide-betaine shows promise as an irrigation solution with good evidence for its efficacy in in-vitro an animal studies but warrants further investigation for use in total joint arthroplasty.

The advantages and disadvantages of different irrigation solutions are summarized (Table 1). This table is not all-inclusive, rather serves as a visual to compare irrigation solutions in the setting of current published literature.

**Table 1.** Comparison of Intraoperative Joint Irrigation Solutions

**Solution**	**Advantages**	**Disadvantages**
Povidone-iodine	Inexpensive, no induced bacterial resistance	Chondrotoxicity at high concentrations, theoretical systemic iodine toxicity
Chlorhexidine	Broad spectrum antibacterial coverage	Chondrotoxicity
Acetic acid	Broad spectrum antibacterial coverage	Poorly studied
Hydrogen peroxide	Appears to be synergistic with other solutions	Wound healing concerns and theoretical risks of increased embolic events
Antibiotic solution	Ability to personalize treatment	No proven efficacy
Taurolidine	Antimicrobial and antifungal properties	No proven efficacy
Polyhexanide-betaine	Broad spectrum, no induced bacterial resistance	Concerns for chondrotoxicity, limited in-vivo evidence

## CONCLUSIONS

Prosthetic joint infections remain a devastating complication in total joint arthroplasty. Irrigation solutions have been increasingly studied as an intraoperative measure to help prevent PJI. The current literature fails to demonstrate a clear consensus for a preferred solution and concentration. Prospective, randomized control trials directly comparing different irrigation solutions are needed. An ideal irrigation solution would be both efficacious and safe. If a solution were discovered or developed that could reliably eradicate bacterial biofilm, the prevention and treatment of PJI could be revolutionized. Current literature of povidone-iodine, chlorhexidine, hydrogen peroxide, acetic acid, antibiotic irrigations, taurolidine, and polyhexanide-betaine are mixed. Further studies, including comparative in-vivo studies, are warranted.

### Conflict of Interest

None that apply to this study.

## References

[ref-137152] Izakovicova Petra, Borens Olivier, Trampuz Andrej (2019). Periprosthetic joint infection: current concepts and outlook. EFORT Open Reviews.

[ref-137153] Driesman Adam, Shen Michelle, Feng James E., Waren Daniel, Slover James, Bosco Joseph, Schwarzkopf Ran (2020). Perioperative chlorhexidine gluconate wash during joint arthroplasty has equivalent periprosthetic joint infection rates in comparison to betadine wash. The Journal of Arthroplasty.

[ref-137154] Sloan Matthew, Premkumar Ajay, Sheth Neil P. (2018). Projected volume of primary total joint arthroplasty in the U.S., 2014 to 2030. Journal of Bone and Joint Surgery.

[ref-137155] Sharkey Peter F., Lichstein Paul M., Shen Chao, Tokarski Anthony T., Parvizi Javad (2014). Why are total knee arthroplasties failing today-has anything changed after 10 years?. The Journal of Arthroplasty.

[ref-137156] Wen-Li D., Ze-Ming L., Zhan-Jun S., Wang J. (2020). Outcomes following revision total knee arthroplasty septic versus aseptic failure: a national propensity-score-matched comparison. J Knee Surg.

[ref-137157] Arciola Carla Renata, Campoccia Davide, Montanaro Lucio (2018). Implant infections: adhesion, biofilm formation and immune evasion. Nature Reviews Microbiology.

[ref-137158] Gbejuade Herbert O, Lovering Andrew M, Webb Jason C (2015). The role of microbial biofilms in prosthetic joint infections. Acta Orthopaedica.

[ref-137159] Urish Kenneth L., DeMuth Peter W., Craft David W., Haider Hani, Davis Charles M., III (2014). Pulse lavage is inadequate at removal of biofilm from the surface of total knee arthroplasty materials. The Journal of Arthroplasty.

[ref-137160] Kildow Beau J., Della-Valle Craig J., Springer Bryan D. (2020). Single vs 2-stage revision for the treatment of periprosthetic joint infection. The Journal of Arthroplasty.

[ref-137161] Calkins Tyler E., Culvern Chris, Nam Denis, Gerlinger Tad L., Levine Brett R., Sporer Scott M., Della Valle Craig J. (2020). Dilute betadine lavage reduces the risk of acute postoperative periprosthetic joint infection in aseptic revision total knee and hip arthroplasty: a randomized controlled trial. The Journal of Arthroplasty.

[ref-137162] von Kuedell Arvind, Canseco Jose A., Gomoll Andreas H. (2013). Deleterious effects of diluted povidone-iodine on articular cartilage. The Journal of Arthroplasty.

[ref-137163] Brown Nicholas M., Cipriano Cara A., Moric Mario, Sporer Scott M., Della Valle Craig J. (2012). Dilute betadine lavage before closure for prevention of acute postoperative deep periprosthetic joint infection. The Journal of Arthroplasty.

[ref-137164] Douw C. M., Bulstra S. K., Vandenbroucke J., Geesink R. G. T., Vermeulen A. (1998). Clinical and pathological changes in the knee after accidental chlorhexidine irrigation during arthroscopy. The Journal of Bone and Joint Surgery. British volume.

[ref-137165] Goswami Karan, Cho Jeongeun, Foltz Carol, Manrique Jorge, Tan Timothy L., Fillingham Yale, Higuera Carlos, Della Valle Craig, Parvizi Javad (2019). Polymyxin and bacitracin in the irrigation solution provide no benefit for bacterial killing in vitro. Journal of Bone and Joint Surgery.

[ref-137166] Schmidt Kenneth, Estes Chris, McLaren Alex, Spangehl Mark J. (2018). Chlorhexidine antiseptic irrigation eradicates Staphylococcus epidermidis from biofilm. An in vitro study. Clinical Orthopaedics & Related Research.

[ref-137167] Tsang S. T. J., Gwynne P. J., Gallagher M. P., Simpson A. H. R. W. (2018). The biofilm eradication activity of acetic acid in the management of periprosthetic joint infection. Bone & Joint Research.

[ref-137168] Ulivieri Simone, Toninelli Stefano, Petrini Carlo, Giorgio Antonio, Oliveri Giuseppe (2011). Prevention of post-operative infections in spine surgery by wound irrigation with a solution of povidone–iodine and hydrogen peroxide. Archives of Orthopaedic and Trauma Surgery.

[ref-137169] Ruder John A., Springer Bryan D. (2017). Treatment of periprosthetic joint infection using antimicrobials: dilute povidone-iodine lavage. Journal of Bone and Joint Infection.

[ref-137170] Bigliardi Paul Lorenz, Alsagoff Syed Abdul Latiff, El-Kafrawi Hossam Yehia, Pyon Jai-Kyong, Wa Chad Tse Cheuk, Villa Martin Anthony (2017). Povidone iodine in wound healing: a review of current concepts and practices. International Journal of Surgery.

[ref-137171] Lacey R.W., Catto A. (1993). Action of povidone-iodine against methicillin-sensitive and -resistant cultures of Staphylococcus aureus. Postgrad Med J.

[ref-137172] Houang E T, Gilmore O J, Reid C, Shaw E J (1976). Absence of bacterial resistance to povidone iodine.. Journal of Clinical Pathology.

[ref-137173] Chundamala J., Wright J.G. (2007). The efficacy and risks of using povidone-iodine irrigation to prevent surgical site infection: an evidence-based review. Can J Surg.

[ref-137174] Gilotra M., Nguyen T., Jaffe D., Sterling R. (2015). Dilute betadine lavage reduces implant-related bacterial burden in a rabbit knee prosthetic infection model. Am J Orthop.

[ref-137175] Cichos Kyle H., Andrews Rachel M., Wolschendorf Frank, Narmore Whitney, Mabry Scott E., Ghanem Elie S. (2019). Efficacy of intraoperative antiseptic techniques in the prevention of periprosthetic joint infection: superiority of betadine. The Journal of Arthroplasty.

[ref-137176] Goldenheim P.D. (1993). In vitro efficacy of povidone-iodine solution and cream against methicillin-resistant Staphylococcus aureus. Postgrad Med J.

[ref-137177] Hernandez Nicholas M., Hart Adam, Taunton Michael J., Osmon Douglas R., Mabry Tad M., Abdel Matthew P., Perry Kevin I. (2019). Use of povidone-iodine irrigation prior to wound closure in primary total hip and knee arthroplasty: an analysis of 11,738 cases. Journal of Bone and Joint Surgery.

[ref-137178] Hart Adam, Hernandez Nicholas M., Abdel Matthew P., Mabry Tad M., Hanssen Arlen D., Perry Kevin I. (2019). Povidone-iodine wound lavage to prevent infection after revision total hip and knee arthroplasty: an analysis of 2,884 cases. Journal of Bone and Joint Surgery.

[ref-137179] Blom Ashley, Cho Jeoung Eun, Fleischman Andrew, Goswami Karan, Ketonis Constantinos, Kunutsor Setor K., Makar Gabriel, Meeker Daniel G., Morgan-Jones Rhidian, Ortega-Peña Silvestre, Parvizi Javad, Smeltzer Mark, Stambough Jeffrey B., Urish Kenneth, Ziliotto Giorgio (2019). General assembly, prevention, antiseptic irrigation solutions: proceedings of international consensus on orthopedic infections. The Journal of Arthroplasty.

[ref-137180] Allegranzi Benedetta, Bischoff Peter, de Jonge Stijn, Kubilay N Zeynep, Zayed Bassim, Gomes Stacey M, Abbas Mohamed, Atema Jasper J, Gans Sarah, van Rijen Miranda, Boermeester Marja A, Egger Matthias, Kluytmans Jan, Pittet Didier, Solomkin Joseph S (2016). New WHO recommendations on preoperative measures for surgical site infection prevention: An evidence-based global perspective. The Lancet Infectious Diseases.

[ref-137181] Berríos-Torres Sandra I., Umscheid Craig A., Bratzler Dale W., Leas Brian, Stone Erin C., Kelz Rachel R., Reinke Caroline E., Morgan Sherry, Solomkin Joseph S., Mazuski John E., Dellinger E. Patchen, Itani Kamal M. F., Berbari Elie F., Segreti John, Parvizi Javad, Blanchard Joan, Allen George, Kluytmans Jan A. J. W., Donlan Rodney, Schecter William P., for the Healthcare Infection Control Practices Advisory Committee (2017). Center for disease control and prevention guideline for the prevention of surgical site infection. JAMA Surgery.

[ref-137182] Hagi Akifumi, Iwata Koushi, Nii Takuya, Nakata Hikaru, Tsubotani Yoshie, Inoue Yasuhide (2015). Bactericidal effects and mechanism of action of olanexidine gluconate, a new antiseptic. Antimicrobial Agents and Chemotherapy.

[ref-137183] McDonnell Gerald, Russell A. Denver (1999). Antiseptics and disinfectants: activity, action, and resistance. Clinical Microbiology Reviews.

[ref-137184] Brennan S.S., Foster M.E., Leaper D.J. (1986). Antiseptic toxicity in wounds healing by secondary intention. Journal of Hospital Infection.

[ref-137185] Han Yung, Giannitsios Demetri, Duke Kajsa, Steffen Thomas, Burman Mark (2011). Biomechanical analysis of chlorhexidine power irrigation to disinfect contaminated anterior cruciate ligament grafts. The American Journal of Sports Medicine.

[ref-137186] Schwechter Evan M., Folk David, Varshney Avanish K., Fries Bettina C., Kim Sun Jin, Hirsh David M. (2011). Optimal irrigation and debridement of infected joint implants: an in vitro methicillin-resistant Staphylococcus aureus biofilm model. The Journal of Arthroplasty.

[ref-137187] Smith Daniel C., Maiman Richard, Schwechter Evan M., Kim Sun Jin, Hirsh David M. (2015). Optimal irrigation and debridement of infected total joint implants with chlorhexidine gluconate. The Journal of Arthroplasty.

[ref-137188] Koo Hyun, Allan Raymond N., Howlin Robert P., Stoodley Paul, Hall-Stoodley Luanne (2017). Targeting microbial biofilms: current and prospective therapeutic strategies. Nature Reviews Microbiology.

[ref-137189] Steinberg D., Heling I., Daniel I., Ginsberg I. (1999). Antibacterial synergistic effect of chlorhexidine and hydrogen peroxide against Streptococcus sobrinus, Streptococcus faecalis and Staphylococcus aureus. Journal of Oral Rehabilitation.

[ref-137190] Frisch Nicholas B., Kadri Omar M., Tenbrunsel Troy, Abdul-Hak Abraham, Qatu Mossub, Davis Jason J. (2017). Intraoperative chlorhexidine irrigation to prevent infection in total hip and knee arthroplasty. Arthroplasty Today.

[ref-137191] Johnston C.S., Gaas C.A. (2006). Vinegar: Medicinal uses and antiglycemic effect. MedGenMed.

[ref-137192] Halstead Fenella D., Rauf Maryam, Moiemen Naiem S., Bamford Amy, Wearn Christopher M., Fraise Adam P., Lund Peter A., Oppenheim Beryl A., Webber Mark A. (2015). The antibacterial activity of acetic acid against biofilm-producing pathogens of relevance to burns patients. PLoS One.

[ref-137193] Williams Rhodri L., Ayre Wayne N., Khan Wasim S., Mehta Amisha, Morgan-Jones Rhidian (2017). Acetic acid as part of a debridement protocol during revision total knee arthroplasty. The Journal of Arthroplasty.

[ref-137194] Thomas E L, Milligan T W, Joyner R E, Jefferson M M (1994). Antibacterial activity of hydrogen peroxide and the lactoperoxidase-hydrogen peroxide-thiocyanate system against oral streptococci. Infection and Immunity.

[ref-137195] George David A., Konan Sujith, Haddad Fares S. (2015). Single-stage hip and knee exchange for periprosthetic joint infection. The Journal of Arthroplasty.

[ref-137196] Zubko Elena I, Zubko Mikhajlo K (2013). Co-operative inhibitory effects of hydrogen peroxide and iodine against bacterial and yeast species. BMC Research Notes.

[ref-137197] Mut Melike, Yemisci Muge, Gursoy-Ozdemir Yasemin, Ture Ugur (2009). Hydrogen peroxide–induced stroke: elucidation of the mechanism in vivo. Journal of Neurosurgery.

[ref-137198] Timperley A.J., Bracey D.J. (1989). Cardiac arrest following the use of hydrogen peroxide during arthroplasty. The Journal of Arthroplasty.

[ref-137199] Siddiqi Ahmed, Forte Salvador A., Docter Shgufta, Bryant Dianne, Sheth Neil P., Chen Antonia F. (2019). Perioperative antibiotic prophylaxis in total joint arthroplasty: a systematic review and meta-analysis. Journal of Bone and Joint Surgery.

[ref-137200] Smith E.B., Wynne R., Joshi A., Liu H., Good R.P. (2012). Is it time to include vancomycin for routine perioperative antibiotic prophylaxis in total joint arthroplasty patients?. J Arthroplasty.

[ref-137201] Anglen Jeff, Apostoles Peter Steven, Christensen Gordon, Gainor Barry, Lane Joel (1996). Removal of surface bacteria by irrigation. Journal of Orthopaedic Research.

[ref-137202] Anglen JEFFREY O. (2005). Comparison of soap and antibiotic solutions for irrigation of lower-limb open fracture wounds: a prospective, randomized study. The Journal of Bone and Joint Surgery-American Volume.

[ref-137203] de Jonge Stijn W., Boldingh Quirine J.J., Solomkin Joseph S., Allegranzi Benedetta, Egger Matthias, Dellinger E. Patchen, Boermeester Marja A. (2017). Systematic review and meta-analysis of randomized controlled trials evaluating prophylactic intra-operative wound irrigation for the prevention of surgical site infections. Surgical Infections.

[ref-137204] Jacobi Christoph A., Menenakos Charalambos, Braumann Chris (2005). Taurolidine–a new drug with anti-tumor and anti-angiogenic effects. Anti-Cancer Drugs.

[ref-137205] Woo Young Ha, Jeong Ju Seon, Kim Ok Geol, Lee In Seung (2018). Efficacy of taurolidine irrigation in primary total knee arthroplasty. Knee Surgery and Related Research.

[ref-137206] Kaehn K. (2010). Polihexanide: a safe and highly effective biocide. Skin Pharmacology and Physiology.

[ref-137207] Davis Stephen J, Harding Andrew T, Gil Joel A, Parajon Fernando, Valdes Jose, Solis Michael, Higa Alex (2017). Effectiveness of a polyhexanide irrigation solution on methicillin-resistant *Staphylococcus aureus* biofilms in a porcine wound model. International Wound Journal.

[ref-137208] Christopher Zachary K., Tran Christine Phuong, Vernon Brent L., Spangehl Mark J. (2022). What is the duration of irrigation? An in vitro study of the minimum exposure time to eradicate bacteria with irrigation solutions. The Journal of Arthroplasty.

